# The effect of BMI on long-term outcomes after operatively treated ankle fractures: a study with up to 16 years of follow-up

**DOI:** 10.1186/s12891-022-05247-3

**Published:** 2022-04-04

**Authors:** Diogo Vieira Cardoso, Joris Paccaud, Victor Dubois-Ferrière, Christophe Barea, Didier Hannouche, Andrea Veljkovic, Anne Lübbeke

**Affiliations:** 1 Division of Orthopaedics and Trauma Surgery, Geneva University Hospitals, Geneva, Switzerland; 2 Footbridge Centre for Integrated Orthopaedic Care, Vancouver, BC Canada; 3 Nuffield Department of Orthopaedics, Rheumatology and Musculoskeletal Sciences, University of Oxford, Oxford, UK

**Keywords:** Ankle fracture, Manchester-Oxford foot and ankle questionnaire (MOXFQ), Obesity, BMI, Danis-Weber, Malleolar

## Abstract

**Background:**

Ankle fractures are a common injury and the main cause of post-traumatic ankle arthritis. The prevalence of obesity is increasing worldwide, and this population is known to have poorer short and midterm outcomes after ankle fractures. Our objective is to assess long-term patient-reported outcomes in patients with operatively treated ankle fractures, and the effect of BMI on these results using the new and validated patient-reported outcome questionnaire, the Manchester Oxford foot and ankle questionnaire (MOXFQ).

**Methods:**

We performed a retrospective review of all ankle fractures treated operatively in a ten-year period from 2002–2012. The MOXFQ and SF-12 were sent to all patients and were obtained, on average, 11.1 years after surgery (range 5.3–16.2 years).

**Results:**

Two thousand fifty-five ankle fractures were reviewed, of which 478 (34%) patients completed the questionnaires. The mean age was 48.1 ± 15.5 years, 52% were men and the mean BMI was 26.1 ± 4.5 kg/m2. Of the 478, 47% were of normal weight, 36% were overweight, and 17% were obese. Overall, 2.1% were type A, 69.9% B, and 24.9% type C fractures. There were no significant differences in the type of fracture between the BMI groups. Comparing obese and non-obese patients, there were large differences in MOXFQ pain (33 ± 29 vs. 18.7 ± 22.1, effect size 0.55), and function scores (27.3 ± 29 vs. 12.5 ± 21.1, effect size 0.58). No differences in complications and reoperations rates were observed. The BMI value at surgery correlated more strongly with the MOXFQ pain score than the BMI at follow-up (Spearman’s Rho 0.283 vs. 0.185, respectively).

**Conclusion:**

These findings reveal that obese patients have significant worse long-term outcomes, namely increased pain, poorer function, and greater impairment in everyday life after an operatively treated ankle fracture. Moreover, pain and function linearly declined with increasing BMI. Our findings appear to indicate that increased BMI at surgery is an important contributor to adverse outcome in the operative management of rotational ankle fractures.

**Level of evidence:**

III.

## Introduction

Ankle fractures in adults are common injuries, accounting for 10% of all fractures. Their incidence has been increasing since the 1950s; a recent study reported an incidence as high as 168.7/100,000 person-years [1, 2]. Population aging, increasing obesity prevalence, and widespread participation in sports activities are considered the major risk factors [3]. A report from the World Health Organization (WHO) stated that the worldwide prevalence of obesity nearly tripled between 1975 and 2016,and 13% of the world adult population were obese [4].

The adverse effects of obesity in the orthopedic field are well known. It has been demonstrated that pain complaints are more prevalent as body mass index (BMI) increases, and the likelihood of having pain in morbidly obese patients is four times higher than among non-obese patients [5].Compared to non-obese patients, the need for a total knee arthroplasty is estimated to be 8.5 times higher in patients with a BMI of 40 or more [6]. Obesity or increasing BMI has been associated with more severe ankle fractures and open fractures [7–9]. Worse short and midterm outcomes after ankle fractures in the obese population have been reported with higher pain scores and worse functional outcomes on several scores, such as the American Orthopaedic Foot and Ankle Society (AOFAS) and the Olerud Molander Ankle Score (OMAS) [8, 10, 11].While their use is widely accepted among orthopedic surgeons, the AOFAS has not been appropriately validated in foot and ankle disorders [12].In addition, these questionnaires are physician-assessed and not patient-reported outcome measures (PROMs).

The Manchester Oxford foot and ankle questionnaire (MOXFQ), a 16-item PROM validated for use in a wide range of foot and ankle pathologies, has been translated and validated for several languages, including French, Italian, Spanish, Turkish, Dutch, German, Persian, and Korean [13–21]. The psychometric properties of the MOXFQ have compared favorably with those of (AOFAS), Self-reported Foot and Ankle Score (SEFAS), Foot and Ankle Outcome Score (FAOS) and the SF-36 [16, 22–25].

As mentioned above, worse short- to mid-term outcomes after malleolar fractures in obese patients have been reported (maximum follow up was six years), but no published data are available on long-term outcomes [11, 26]. Moreover, while the relationship between obesity and poor outcomes in orthopedic patients is established, the question of whether this is due to their being obese at the time of follow-up or at the time of the injury/surgery or both is still unanswered. Our objective was to evaluate long-term patient-reported outcomes in patients with operatively treated ankle fractures, and to assess the effect of BMI on their outcome using the new and validated patient-reported questionnaire MOXFQ.

## Material and methods

### Patients

The local ethics committee approved this study. We performed an electronic chart review of every consecutive operatively treated rotational ankle fracture in patients aged 18 years old or more between January 2002 to December 2012. The hospital is the largest level-1 trauma center.

All rotational ankle fractures treated with open reduction and internal fixation (ORIF) were identified using the discharge diagnoses recorded in the hospital’s information system. ORIF was performed by a trauma senior staff or a senior resident in in conjunction with a senior staff. Data on patient’s baseline characteristics, including age, sex, comorbidities (American Society of Anesthesiologists (ASA) score, and diabetes), and lifestyle factors (BMI and smoking status) were obtained from the anesthesia records. BMI was calculated by dividing patients’ weight (kg) by their height (m^2^) and classified in four categories (underweight, normal, overweight and obese) according WHO [27]. Mechanisms of accidents were obtained from the emergency reports. Fracture were classified using the Danis–Weber classification in type A or infrasyndesmotic ( OTA/OA 44-A), type B or transyndesmotic ( OTA/OA 44-B) and type C or suprasyndemotic (OTA/OA 44C) based on preoperative radiographs. Fractures were further classified according to the Broos and Bisschop’s classification [28].This classification classifies fractures into unimalleolar, bimalleolar et trimalleolar according the number of fractured malleolus. The first author reviewed all the radiographs, evaluating fracture patterns, and fracture displacement on the preoperative radiograph. Fracture displacement was defined as a gap of 6 mm or more between fragments measured on ankle x-ray. We chose 6 mm of displacement to have a superior displacement of that tradionatlly used by the surgeons when deciding to perform an ORIF. Historically, 2 mm of displacement was one of the mains factors deciding operative treatment vs. conservative treatment for ankle fractures [29]. To assess the interobserver reliability of the classification system, an independent analysis of 105 randomly selected cases was performed.

### Complications and reoperation rates

Incidence of postoperative complications that needed surgical intervention was assessed using the patient's chart and postoperative radiographs and was divided as follows: 1) wound healing problems including dehiscence and wound infection; 2) neurovascular impairment; 3) fracture nonunion; 4) hardware fixation failure with loss of reduction. Reoperation for ankle arthrodesis, ankle replacement and impingement ressection were identified. Postoperative hardware-related pain and additional surgery performed for hardware removal was also noted.

### Questionnaires

The questionnaires that were sent out to the patients and included the MOXFQ and the SF-12, as well as a question about the patient’s current weight and height.

The MOXFQ is a patient-administered measure consisting of 16 items. The recently translated and validated French version was used [20]. The questionnaire measures three domains: walking/standing (7 items), pain (5 items), and social interaction (4 items). Each item is scored from zero to four, with four representing the worst state. The scores for each domain are calculated by averaging the responses to each item within a given domain. The scores were transformed to a zero to 100 scale, with 100 indicating the worst possible condition. The three domains were summed to provide a single summary index score [30]. The questionnaires were sent in early 2019 by mail to all eligible patients (patients still alive and with a known address).

The SF-12 is a self-reported outcome measure assessing the impact of health on an individual's everyday life. Physical and mental health composite scores (PCS and MCS) are computed using the scores of twelve questions and range from zero to 100, where a zero score represents the lowest level of health.

### Statistical analysis

First, baseline characteristics were assessed for all patients operated between 2002 and 2012 and for those who had responded to the questionnaire at follow-up in order to detect substantial discrepancies. In the latter group baseline characteristics were also assessed for each BMI category (BMI at surgery) separately. Second, MOXFQ questionnaire results at follow-up were presented by domain and with the Single Index Score. Unadjusted and adjusted mean differences between the normal weight and the obese group were obtained using a general linear model. Adjustment was performed for potential covariate variables that differed substantially between the categories and those we felt were relevant based on a priori knowledge (age, ASA score, smoking status, diabetes, displaced fracture yes/no). Third, effect sizes (Cohen’s d) of the score differences between normal-weight and obese patients were calculated to evaluate their clinical importance. Effect sizes of 0.2, 0.5, and 0.8 were regarded as a small, medium, and large degrees of difference, respectively [31]. Moreover, minimally important changes (in the context before/after foot or ankle surgery) have been published for the MOXFQ domains [24]. We evaluated our results with regard to those values. Fourth, in the subgroup of patients with information on both BMI at surgery and BMI at follow-up, we separately evaluated their influence on the MOXFQ domains using with the Spearman’s rank correlation coefficient. The latter was chosen because, in contrast to the normally distributed variables for BMI at the time of surgery and follow-up, the distribution of the MOXFQ sub-scores’s was right skewed and considered non-normal.

## Results

**Fig. 1:**
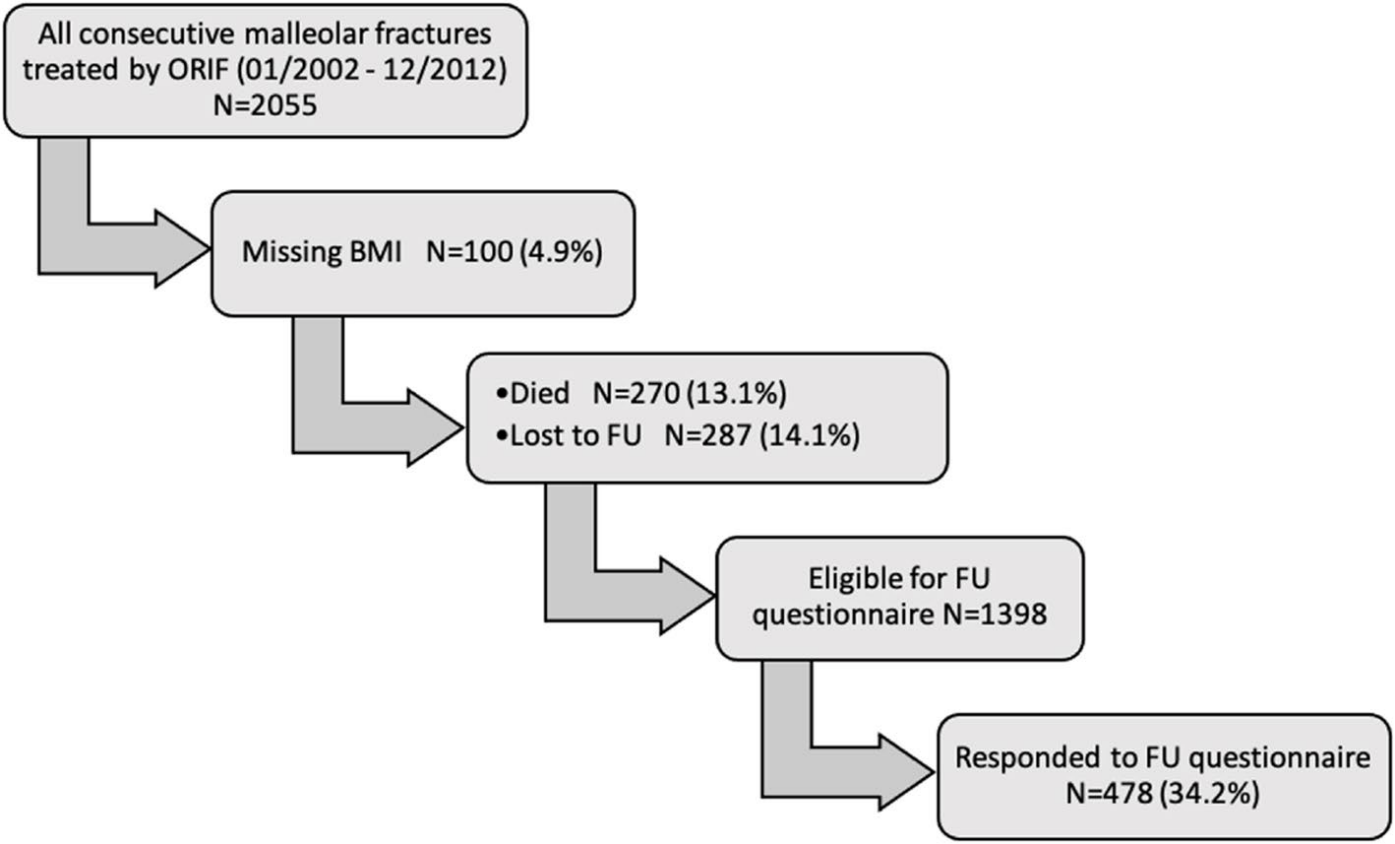
Flowchart. *ORIF* open reduction internal fixation, *BM*I Body mass index, *FU* Follow-up

Over the period, the trauma center operatively treated 2055 ankle fractures that were deemed appropriate operative candidates based on fracture pattern. Of these, 1398 patients were eligible for the follow-up questionnaire, and of those, 478 (34.2%) completed the questionnaire (Fig. 1).

Of the 478 patients, who had responded to the questionnaire, 248 (51.9%) were men (Table 1). The mean patient age was 48.1 ± 15.5 years. The mean BMI was 26.1 ± 4.5 kg/m^2^. Of all, 226 (47.3%) were normal weight, 170 (35.6%) were overweight, and 82 (17.2%) were obese*.* Obese patients had higher ASA scores (*P* < 0.001) and were more often diabetic (*P* = 0.004) and never smokers (*P* = 0.012). According to the Danis-Weber classification, when broken down by type of fracture, 2.1% were type A, 69.9% type B, and 24.9% type C fractures. No large differences were seen in the type of accident and fracture except for a higher proportion of displaced fractures in obese vs. normal-weight patients (45.1% vs. 33.2%; *p* = 0.054). Interobserver reliability of two independent reviewers in classifying a random sample of 105 x-ray cases resulted in almost perfect agreement, with an ICC of 0.894 (95% CI 0.844 to 0.928).

**Table 1:** Baseline characteristics of the patients initially operated and those responding to MOXFQ (all and by BMI category)

	**Operated**	**Responders all**	**Normal weight**	**Overweight**	**Obese**
**Patients**	2055	478	226	170	82
**Age (years), mean (SD)**	49.2 (± 18.3)	48.1 (± 15.5)	46.6 (± 16.2)	49.1 (± 15)	49.9 (± 14.2)
**Men (%)**	1036 (50.4)	248 (51.9)	116 (51.3)	93 (54.7)	39 (47.6)
**Lifestyle factors**					
** BMI (kg/m**^**2**^**), mean (SD)**	26.2 (± 4.9)	26.1 (± 4.5)	22.4 (± 1.8)	27.4 (± 1.4)	33.5 (± 3.1)
BMI by category (%)					
Normal weight (18.5–24.9)	887 (45.4)	226 (47.3)			
Overweight (25–29.9)	691 (35.3)	170 (35.6)			
Obese (≥ 30)	377 (19.3)	82 (17.2)			
**Smoking status (%)**					
Current smoker	710 (34.5)	145 (30.3)	74 (32.7)	55 (32.3)	16 (19.5)
Former smoker	164 (8.0)	40 (8.4)	12 (5.3)	15 (8.8)	13 (15.9)
Never smoked	898 (43.7)	237 (49.6)	110 (48.7)	79 (46.5)	48 (58.5)
Not known if ever smoked	283 (13.8)	56 (11.7)	30 (13.3)	21 (12.4)	5 (6.1)
**Comorbidities**					
** Diabetes (%)**	150 (7.4)	25 (5.2)	6 (2.7)	9 (5.3)	10 (12.2)
**ASA score (%)**					
I	699 (34.1)	194 (40.6)	109 (48.2)	72 (42.4)	13 (15.9)
II	1137 (55.5)	273 (57.1)	116 (51.3)	91 (53.5)	66 (80.5)
III	213 (10.4)	9 (1.9)	1 (0.4)	6 (3.5)	2 (2.4)
Missing	6 (0.3)	2 (0.4)	0	1 (0.6)	1 (1.2)
**Fracture characteristics**					
** Right side (%)**	1103 (53.8)	250 (52.3)	119 (52.7)	93 (54.7)	38 (46.3)
** Open fracture (%)**	157 (7.7)	30 (6.3)	17 (7.5)	10 (5.9)	3 (3.7)
** Displaced fracture (%)**	683 (36.6)	172 (36.0)	75 (33.2)	60 (35.3)	37 (45.1)
**Danis-Weber classification (%)**					
A	48 (2.3)	10 (2.1)	6 (2.7)	3 (1.8)	1 (1.2)
B	1492 (72.6)	334 (69.9)	155 (68.6)	120 (70.6)	59 (72.0)
C	410 (20.0)	119 (24.9)	54 (23.9)	44 (25.9)	21 (25.6)
Non-classifiable	105 (5.1)	15 (3.1)	11 (4.9)	3 (1.8)	1 (1.2)
**Number fractured malleoli (%)**					
Unimalleolar	1110 (54.0)	257 (53.8)	128 (56.6)	88 (51.8)	41 (50.0)
Bimalleolar	511 (24.9)	104 (21.8)	44 (19.5)	40 (23.5)	20 (24.4)
Trimalleolar	433 (21.1)	116 (24.3)	53 (23.5)	42 (24.7)	21 (25.6)
Missing	1 (0)	1 (0.2)	1 (0.4)	0	0
**Type of accident (%)**					
** Simple fall**	1303 (63.4)	289 (60.5)	130 (57.5)	102 (60.0)	57 (69.5)
** Transportation**	332 (16.2)	94 (19.7)	46 (20.4)	35 (20.6)	13 (15.9)
** Sports**	293 (14.3)	67 (14)	35 (15.5)	25 (14.7)	7 (8.5)
** Other**	127 (6.2)	28 (5.9)	15 (6.6)	8 (4.7)	5 (6.1)

Overall, there were 34 (7%) complications. Of these, 13 (6%) in the normal weight group, 14 (8%) in the overweight group, and 7 (9%) in the obese group (*P* = 0.311) A summary of complications and additional surgeries by BMI categories is illustrated in Table 2. No significative differences in reoperation rates were observed between groups.

**Table 2:** Complications and reoperations by BMI category

	**All**	**Normal weight**	**Overweight**	**Obese**	***P values***
**Complications (%)**	34 (7)				
Wound healing problems	19 (4)	7 (3)	7 (4)	5 (6)	.242
Nonunion	5 (1)	1 (1)	3 (2)	1 (1)	.363
Loss of reduction	10 (2)	5 (2)	4 (2)	1 (1)	.672
**Reoperations (%)**	12 (3)				
Hardware removal (%)	206 (43)	100 (44)	75 (44)	31 (38)	.408
Ankle arthrodesis	5 (1)	2 (1)	2 (1)	1 (1)	.760
Ankle replacement	3 (1)	1	1	1	.482
Impingement	4 (1)	2 (1)	2 (1)	0	.592

The 478 patients who responded to the questionnaire did not substantially differ in baseline characteristics from the 2055 consecutive patients operated between 2002 and 2012, except for a lower proportion in ASA class 3, which is likely related to their higher risk of dying during the long follow-up period.

**Fig. 2:**
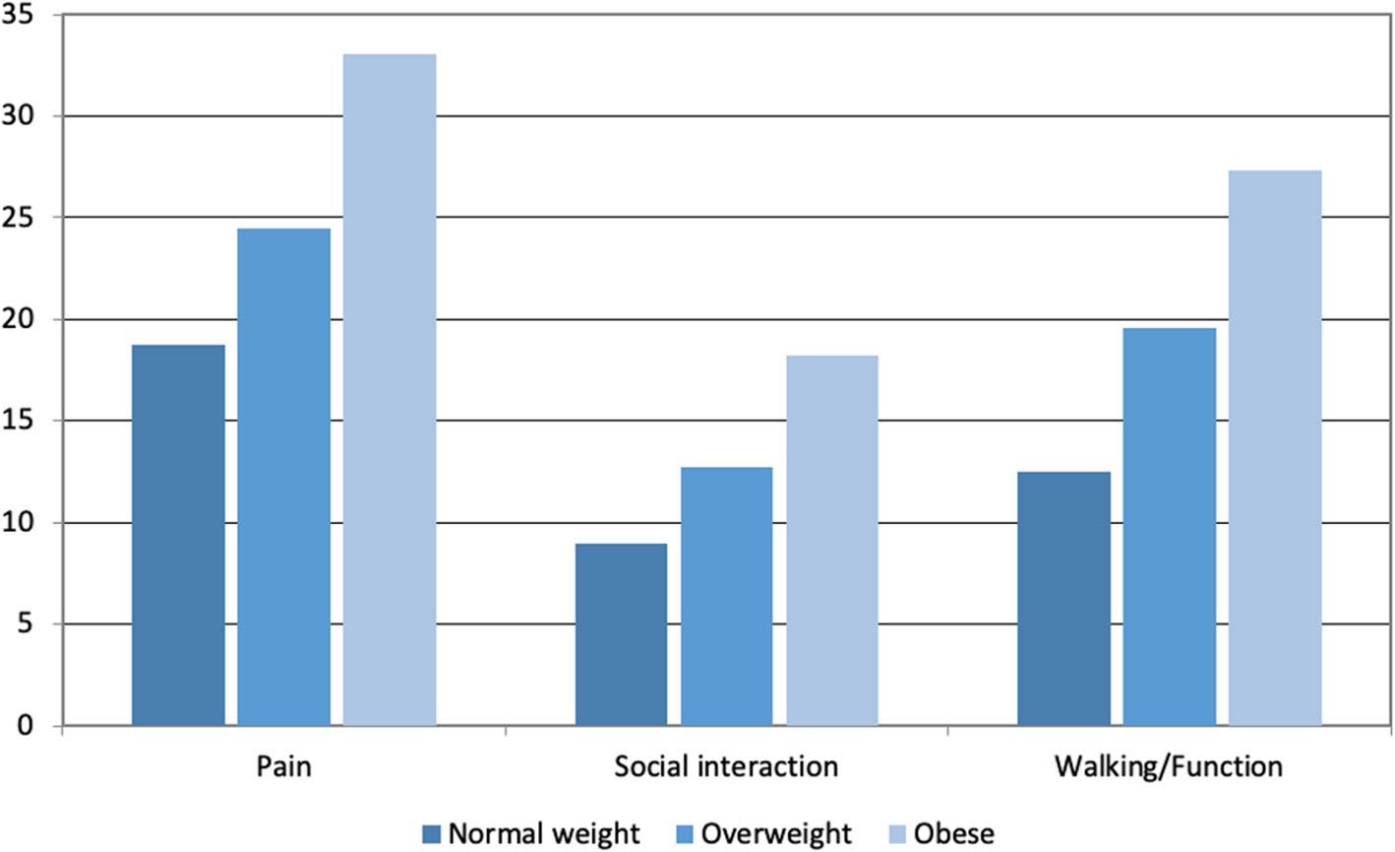
Manchester Oxford Foot Questionnaire by domain and BMI category

**Fig. 3:**
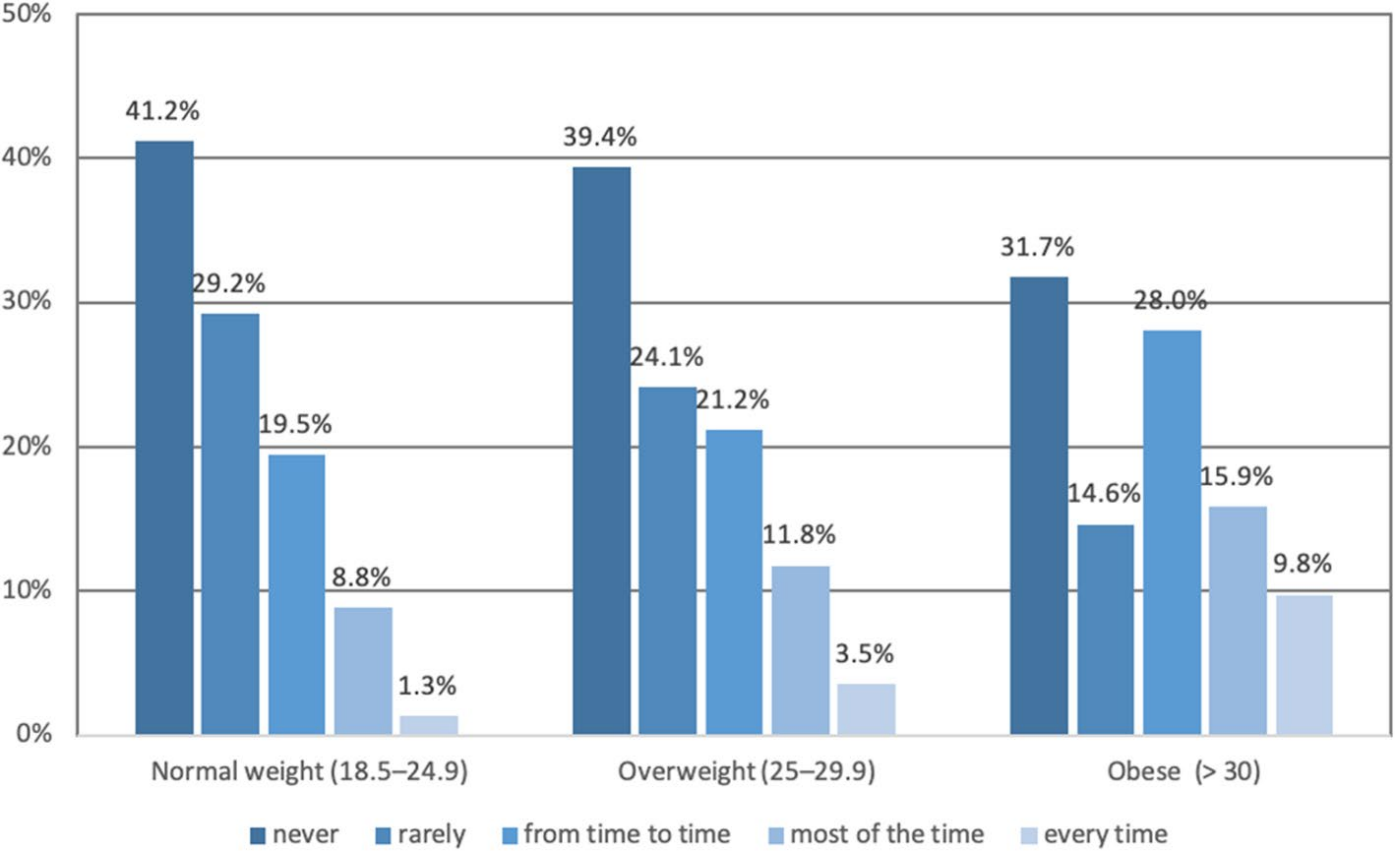
Manchester Oxford Foot Questionnaire Responses item 1 « I have pain in my foot/ankle» by BMI category

**Fig. 4:**
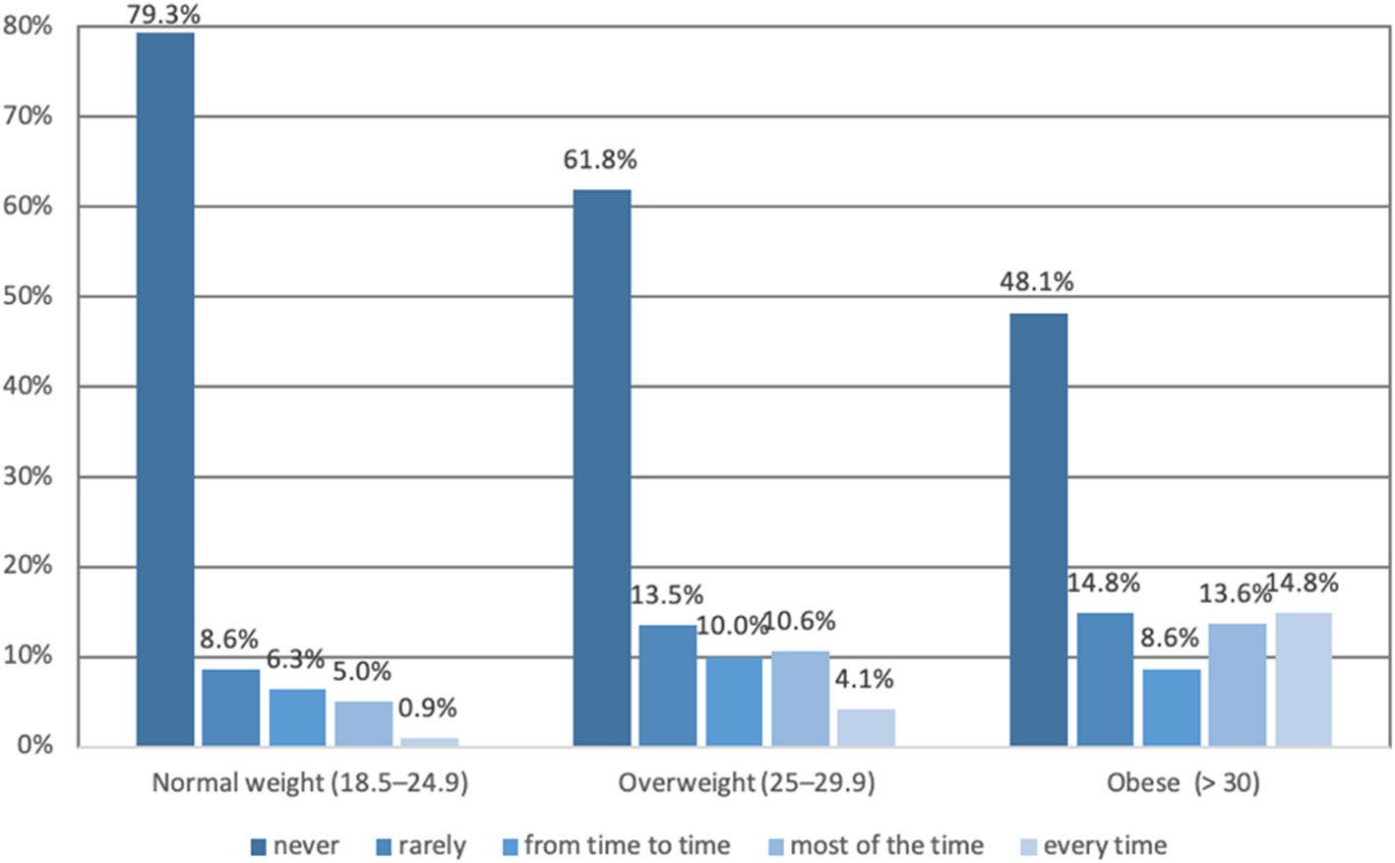
Manchester Oxford Foot Questionnaire responses item 8 «I catch the bus or use the car instead of walking» by BMI category

**Fig. 5:**
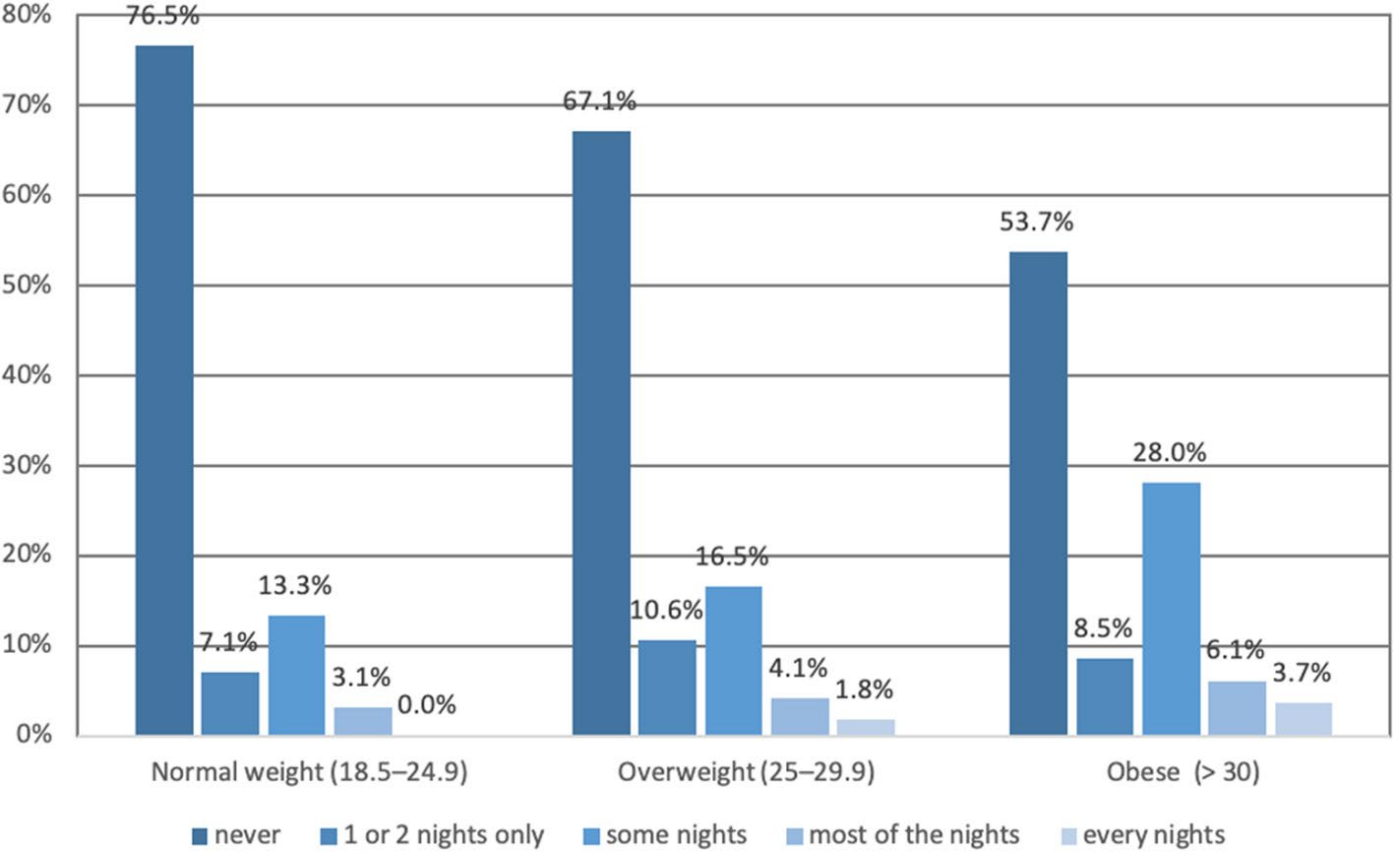
Manchester Oxford Foot questionnaire responses item 16 «During the past 4 weeks, have you been troubled by the pain from your foot in bed at night?» by BMI category

MOXFQ and SF-12 score results obtained, on average, 11.1 years after surgery (range 5.3–16.2 years), linearly worsened with increasing BMI category (Fig. 2). Of the 226 with normal weight, 70% had occasional pain, 20% moderate, and 10% severe pain. In overweight (170) patients, 64% had occasional pain, 21% moderate, and 15% severe pain. In the obese patients (82), 46% had occasional pain, 28% moderate, and 26% severe pain. Pain distribution according to BMI categories is represented in Fig. 3. Figures 4 and 5 illustrate the substantial differences between BMI categories in both the use of bus/car instead of walking due to pain in the foot or ankle, and in the presence of night pain, with obese patients reporting higher levels of pain in both domains.

Comparing obese and non-obese patients, there were significant differences in pain (33 ± 29 vs. 18.7 ± 22.1) and function (27.3 ± 29 vs. 12.5 ± 21.1), (Table 3). There were large statistically significant differences between normal-weight and obese patients on all MOXFQ and SF-12 sub-scores in the unadjusted analyses. Adjustment for baseline imbalances in age, ASA score, smoking status, diabetes, and fracture displacement only marginally lowered the observed differences in the MOXFQ sub-scores and the SF-12 PCS. Effect sizes of the score differences between normal-weight and obese patients were 0.55 for the MOXFQ pain score, 0.58 for walking, 0.44 for social interaction, and they were 0.51 for SF-12 PCS and 0.26 for SF-12 MCS. These effect sizes indicate that the differences were perceivable for the patients, and of moderate clinical importance except for the SF-12 MCS, which was of small clinical importance. The change values (or differences) between obese and non-obese patients were walking 12.7, pain 12.9 and social interaction 7.7 points after adjusting for baseline characteristics (Table 3). This corresponds to clinically relevant differences regarding “walking” and “pain”.

**Table 3:** Manchester Oxford Foot Questionnaire (MOXFQ) and SF-12 by BMI category

	**Responders all**	**Normal weight**	**Overweight**	**Obese**	**Unadjusted Difference (95%CI)**^a^	**Adjusted Difference (95%CI)**^a^
	*N* = 478	*N* = 226	*N* = 170	*N* = 82		
**MOXFQ mean, SD**						
** Pain**	23.2 ± 25.7	18.7 ± 22.1	24.5 ± 27	33 ± 29	14.3 (7.9; 20.7)	12.9 (6.2; 19.5)
** Walking function**	17.6 ± 25.3	12.5 ± 21.1	19.6 ± 26.9	27.3 ± 29	14.8 (8.5; 21.2)	12.7 (6.0; 19.3)
** Social interaction**	11.9 ± 20.4	8.9 ± 17.8	12.8 ± 21	18.3 ± 24.2	9.3 (4.1; 14.6)	7.7 (2.2; 13.2)
** Single index score**	17.8 ± 22.7	13.5 ± 19.3	19.5 ± 24.2	26.3 ± 25.4	12.7 (6.9; 18.6)	11.3 (5.3; 17.3)
** SF-12 mean, SD**	*N* = 456	*N* = 218	*N* = 163	*N* = 75		
** pcs**	47.2 ± 9.6	48.7 ± 9.3	46.7 ± 9.5	43.9 ± 9.7	-4.8 (-2.3; -7.2)	-3.5 (-1.0; -6.0)
** mcs**	46.8 ± 10.4	47.2 ± 10.3	47.3 ± 10.1	44.4 ± 11.1	-2.8 (-0.1; -5.5)	-2.2 (0.6; -5.1)

^a^Unadjusted and adjusted mean differences between normal weight and obese group. Adjustment was performed for age, ASA score, smoking status, diabetes, and fracture displacement using a general linear model

^b^SF-12 *pcs*   physical component score, *mcs*   mental component score

In a subgroup analysis, we included only patients for whom BMI at the time of the follow-up questionnaire was available in addition to the BMI at the time of surgery. Of the 224 patients who had information on both (BMI at surgery and follow-up), 93 (41%) were of normal weight, 87 (39%) overweight, and 44 (20%) were obese at surgery—similar to the distribution in the entire cohort (N = 478). They were also similar to the entire cohort concerning mean age (48 vs. 48.1 years) and sex distribution (52% vs. 55% men). At follow-up, 77 were of normal weight, 87 overweight, and 60 were obese. Overall, in 154 patients (68.8%), the BMI category did not change between surgery and the follow-up 7–16 years later. We evaluated whether the observed differences in the MOXFQ were related to the BMI at the surgery or the BMI at follow-up or both. Both BMI values partly explained variation in outcomes, and correlation coefficients were small to fair. Spearman’s rank correlation coefficients were higher for BMI at surgery for all the domains except for Social Interaction (Table 4). The most considerable difference in the coefficients was seen for pain, with the BMI value at surgery correlating more strongly with the MOXFQ pain score than the BMI at follow-up (Spearman’s Rho 0.283 vs. 0.185, respectively) followed by MOXFQ walking function score (Spearman’s Rho 0.284 vs. 0.194, respectively).

**Table 4:** Correlation of BMI at surgery and BMI at follow-up with the Manchester Oxford foot questionnaire scores (MOXFQ), (= 224 patients*)

	**BMI at surgery**	**BMI at follow-up**
	**Spearman’s rank correlation coefficient**	**Spearman’s rank correlation coefficient**
**MOXFQ**		
** Pain**	0.283	0.185
** Walking function**	0.284	0.194
** Social interaction**	0.251	0.227
** Single index score**	0.288	0.210

^*^Of the 224 patients who had information on both the BMI at surgery and follow-up, 93 were of normal weight, 87 overweight and 44 obese at surgery, and at follow-up 77 were of normal weight, 87 overweight and 60 were obese

## Discussion

The present study aimed to evaluate the effect of BMI on long-term patient-reported outcomes in patients with operatively treated ankle fractures. Our findings reveal that obese patients presented substantially worse long-term outcomes than non-obese patients. These results were mainly observed for the pain and function domains of the MOXFQ questionnaire. Results obtained from the SF-12 also revealed significant differences between both groups, showing that physical disability related to their ankle problems substantially impairs obese patients’ everyday life. Our results are in accordance with the findings of previous shorter-term studies. Stavem et al. found that obese patients had worse scores on the Olerud and Molander Ankle Score (*P* < 0.001), Self-Reported Foot and Ankle Questionnaire (*P* = 0.003) and Lower Extremity Functional Scale (*P* = 0.01) three to six years after ankle surgery than those with normal weight [26]. A recent study published by Benedick et al. involving 632 patients with operatively treated ankle fractures and a mean follow-up of 6.2 years found that total foot function index scores were worse for obese patients [11]. Subcategory scores for disability and activity limitations were worse in the obese group, and obesity was associated with worse SMFA (Short Musculoskeletal Function Assessment) bothersome and mobility scores. The results are consistent with ours. However, none of these previous studies had a follow-up for longer than six years.

The association between obesity and pain is well supported by a large volume of evidence [5, 6, 32, 33].It has been demonstrated that pain complaints are more prevalent as BMI increases, and the likelihood of morbidly obese patients having pain is four times higher than nonobese patients [33]. There are several non-exclusive potential mechanisms for the underlying obesity-pain relationship. Firstly, the mechanical overloading of the joint and increased forces traveling through the joint may cause substantial changes in the cartilage inducing the development of post-traumatic osteoarthritis. One radiographical analysis of osteoarthritic knees in obese patients revealed lower cartilage widths in knee joint space [32]. Secondly, multiple inflammatory markers are elevated in the serum of obese patients as compared to normals, such as interleukin 6, C-reactive protein, and leptin [34, 35]. There is evidence that such endocrine changes may play a role in altered pain modulation in obesity [32]. Thirdly, psychological factors such as depression may play a role in maintaining and potentially facilitating ongoing obesity and pain [32].

Although substantial evidence for the link between joint pain and obesity is available, the literature on obesity and ankle fracture outcomes is scarce. The available data shows that worse outcomes increase with BMI, mainly in pain and function domains [11, 26]. Our results ilustrate that these worse outcomes increase linearly with BMI. However, it is not fully understood if pain complaints and worse functional outcomes result from the ankle fracture, high BMI, or both and whether it is the BMI at the time of surgery or the BMI at follow-up that matters more.

Our cohort analysis suggests that a patient's BMI at the moment of the fracture is an important contributing factor to obese patients' poor outcomes and maybe even more important than BMI at follow-up. The study of Louer et al. allows to better understand the biology and the biochemical effects of obesity in fracture healing [36]. The authors compared serum and degenerative changes in fractured knees in mice on a high-fat diet (obese) to those with a low-fat diet (non-obese). They demonstrated that the high-fat diet increased the serum concentrations of interleukin-12p70, interleukin-6, and keratinocyte-derived chemokine in mice with a joint fracture. Moreover, the same study concluded that the fractured knee joints of the high-fat diet mice showed significantly increased osteoarthritic degeneration compared to the non-fractured contralateral joints. Cytokines including interleukins have been described by others to act as pro-inflammatory mediators causing joint damage and subsequent osteoarthritic degeneration in the ankle [37, 38]. Whether cytokine absolute levels and degree of persistence after ankle fracture depend on BMI has not been studied in humans. Support for a higher degree of osteoarthritic changes in obese possibly related to the poorer clinical outcomes in our study comes from a study by Lübbeke et al. who reported a higher incidence of advanced radiographic osteoarthritis 18 years after operatively treated malleolar fracture in patients overweight or obese at the time of surgery [39].

Our study was not without limitations. Medical records were utilized to collect data retrospectively. Moreover, high patient mobility related to the relatively young age of patients undergoing this type of surgery and the fact that our hospital is a reference trauma center in a tourist area may have contributed to the 34% response rates to our questionnaire. We cannot exclude that in some cases the patient’s decision to participate or not might have been related to the outcome, which could have biased our results. Nevertheless, baseline characteristics were largely similar between all patients who underwent ORIF and those who responded to the follow-up questionnaire.

In conclusion, this study establishes an association between increasing BMI and poor long-term outcomes, namely increased pain, poor function, and impairment in everyday life after an operatively treated ankle fracture. To the best of our knowledge, this is the first study assessing the long-term outcome of ankle fractures in obese patients (average follow-up 11 years). Moreover, it is the first long-term study indicating that elevated BMI at the moment of the fracture is an important contributing factor to obese patients' poor outcomes subsequent to rotational ankle fracture and it may play a more important role than BMI at the follow-up. Given the increasing prevalence of obesity in recent years, these results may help surgeons counsel their patients regarding the outcomes after ankle fracture surgery and set reasonable expectations. Finally, more research on the influence of obesity on fracture healing and potential perioperative treatments is warrant.

## Data Availability

The datasets used and analysed during the current study are available from the corresponding author on reasonable request.

## Declarations

### Methodology

Methods were carried out in accordance with relevant guidelines and regulations.
